# Dynamic patterns of verbal memory function after an initial decline following temporal lobe resection against epilepsy: Sex‐specific differences in the postoperative course

**DOI:** 10.1002/epi.70144

**Published:** 2026-02-14

**Authors:** Pia Langenberg, Lea M. Reisch, Johanna L. Hopf, Florian J. Mücke, Lea Wemheuer, Christian G. Bien, Philip Grewe

**Affiliations:** ^1^ Bielefeld University, Medical School OWL Clinical Neuropsychology and Epilepsy Research Bielefeld Germany; ^2^ Bielefeld University, Medical School OWL Medical Psychology Bielefeld Germany; ^3^ Bielefeld University, Medical School OWL Bethel Epilepsy Center, Department of Epileptology, Krankenhaus Mara Bielefeld Germany; ^4^ Radboud University Nijmegen Donders Institute for Brain, Cognition, and Behaviour, Department of Neuropsychology and Rehabilitation Psychology Nijmegen the Netherlands

**Keywords:** epilepsy surgery, neuropsychology, postoperative outcome, sex differences, verbal memory

## Abstract

**Objective:**

This study was performed to investigate the postoperative dynamics of verbal memory function after temporal lobe resection (TLR) in people with temporal lobe epilepsy (PwTLE), with a specific focus on the course following an initial verbal memory decline and the factors influencing later changes.

**Methods:**

Verbal memory performance of 169 PwTLE was retrospectively analyzed at three time points: preoperatively (T1), 6 months postoperatively (T2), and 24 months postoperatively (T3). At the group level, repeated measures analysis of variance (RM‐ANOVA) was used to evaluate differences in verbal memory across time points between patients who underwent resection in the language‐dominant hemisphere and language‐nondominant hemisphere. At the individual level, frequencies of significant verbal memory changes between time points were calculated. Subsequently, factors influencing late changes (T2–T3) after an initial decline (T1–T2) were analyzed for the language‐dominant resected group.

**Results:**

At the group level, RM‐ANOVA revealed a significant interaction effect (*p* < .001) between time point and resected hemisphere, with significant declines observed in the language‐dominant resected group between T1 and T2 (*p* < .001) and between T1 and T3 (*p* < .001). At the individual level, dynamic patterns of improvement, stability, and decline were observed across time points. In language‐dominant resected PwTLE with an initial decline, RM‐ANOVA showed a significant interaction between time point and sex (*p* < .001); at T3, females exhibited better recovery of verbal memory function than males (*p* = .04).

**Significance:**

These findings underscore the importance of considering individual verbal memory trajectories following TLR, particularly after an initial decline. Notably, sex emerged as a significant factor influencing late postoperative recovery, with women showing greater verbal memory improvement than men. This suggests preliminary evidence for sex‐dependent differences in cognitive recovery and highlights the need for personalized counseling in presurgical decision‐making for PwTLE.

**Plain Language Summary:**

We investigated early and late changes in verbal memory following surgery to treat temporal lobe epilepsy. Our focus was on people who experienced an early memory decline after surgery and how their memory changed over time. We found that patients who had surgery on the language‐dominant side of the brain showed significant early memory decline. However, memory changes after surgery varied greatly from person to person. Notably, women showed better late recovery in memory performance after an early decline than did men. These findings support more refined counseling and personalized care for patients before and after temporal lobe surgery.

**Key points**

- Initial and late memory changes after temporal lobe epilepsy surgery were examined, focusing on the course following an initial decline.
- At the group level, an initial verbal memory decline was found in the language‐dominant resected group, with no further changes over time.
- By contrast, individual‐level analyses revealed dynamic patterns of verbal memory, including improvement, stability, and decline.
- Following language‐dominant resection, females showed greater recovery after an initial decline compared to males.

## 1 INTRODUCTION

Resective surgical treatment can lead to seizure freedom or a significant reduction in seizure frequency for a substantial number of people with temporal lobe epilepsy (PwTLE).^1^ Nevertheless, despite its efficacy, temporal lobe resection (TLR)—particularly in the language‐dominant hemisphere—carries a risk of verbal memory decline,^2^ given the medial temporal lobe's critical role in declarative memory processing.^3^ These memory impairments contribute to diminished quality of life^4^, ^5^ underscoring the broader impact of TLR on daily functioning and well‐being.

Most studies examining decline in verbal memory function after TLR focus on a 6–12‐month follow‐up period, commonly referred to as the initial postoperative phase.^6^, ^7^ Fewer studies have investigated verbal memory functions after longer follow‐up periods (see overview in Baxendale^7^). Rausch et al.^8^ considered both initial and late memory changes after TLR in a longitudinal study examining the postoperative course of 44 PwTLE at the group level, utilizing three measurement points over a period exceeding 9 years. The findings illustrated that an initial verbal memory decline 1 year after left TLR can be followed by a subsequent late decline. By contrast, other studies have shown more stable verbal memory function after an initial decline, between 2 and 10 years post‐TLR.^6^, ^9^ Helmstaedter et al.^10^ found that at the group level, individuals with TLR and ongoing seizures experienced a verbal memory decline over 5–22 years, whereas the seizure‐free cohort maintained stable verbal memory during this period. Notably, at the individual level, among those with ongoing seizures and left TLR, 34% experienced verbal memory decline and 5% improved during the initial postoperative phase; at long‐term follow‐up, 11% demonstrated a late decline, whereas 5% showed a late improvement.

The existing literature indicates that PwTLE may experience an initial decline in verbal memory following resection in the language‐dominant hemisphere, with some studies also suggesting subsequent alterations in the late postoperative course. However, the exact dynamics of verbal memory functions in PwTLE following an initial memory decline, as well as the factors influencing these dynamics, have not been adequately explored. Studies extending beyond the initial postoperative period often show considerable variability in follow‐up intervals and are characterized by small, heterogeneous samples. Additionally, the analysis of meaningful subgroups and case studies could significantly enhance the understanding of individual trajectories compared to group‐level studies.^11^, ^12^

In the current study, we investigated the postoperative dynamics of verbal memory function, with a particular focus on potential changes following an initial postoperative verbal memory decline. Our aim was to provide a more comprehensive understanding of the longer term course of verbal memory function after TLR. To this end, verbal memory performance was examined at multiple structured time points post‐TLR in PwTLE, with attention to both group and individual trends. Given the increased risk of verbal memory decline observed in PwTLE following TLR in the language‐dominant hemisphere,^13^, ^14^ our investigation primarily focused on this cohort. To ensure the specificity of our findings, we also included a group of PwTLE who underwent resection in the language‐nondominant hemisphere.

We hypothesized that there would be a greater initial decline in verbal memory in the language‐dominant resected group than in the language‐nondominant resected group. Building on the expected initial decline in the language‐dominant resected group, we hypothesized that this would be followed by more stable verbal memory performance on the group level, whereas individual trajectories would exhibit fluctuations, with further late decline and also improvement. Consequently, we investigated the influence of well‐established clinical and demographic factors on these late declines or improvements and particularly sought to test the hypotheses that later postoperative change in language‐dominant resected PwTLE and an initial decline would be negatively affected by (1) less postoperative reduction of antiseizure medication,^15^ (2) ongoing seizures,^10^, ^16^ (3) resection of the hippocampus,^2^, ^16^ (4) mesial temporal sclerosis (MTS)^6^ upon histopathological examination or preoperative magnetic resonance imaging (MRI), (5) later age at onset,^17^, ^18^ and (6) male sex.^19^, ^20^, ^21^

## 2 MATERIALS AND METHODS

### 2.1 Participants and procedures

We enrolled all German‐speaking PwTLE who underwent TLR at the Bethel Epilepsy Center between 2014 and 2021 and who completed a neuropsychological assessment preoperatively (T1), as well as 6 (T2) and 24 months (T3) postoperatively. PwTLE with missing neuropsychological data at any of these time points (*n* = 46) were excluded from the dataset. We conducted comparative analyses between PwTLE with complete longitudinal data (completers) and those with missing Verbal Learning and Memory Test (VLMT) assessments at one or more time points (noncompleters), focusing on preoperative verbal memory performance as well as relevant demographic and clinical variables (see Tables S1 and S2). Further exclusion criteria were age of <14 years at T1, the presence of intellectual or learning disability, and atypical or inconclusive language dominance. Language dominance was determined by functional MRI based on a verbal fluency task that previously has been validated with results of a Wada test paradigm.^22^ In two cases, language dominance was determined by results of the Wada test only, due to inconclusive functional MRI findings. A flowchart of the inclusion and exclusion criteria can be found in Figure S1. Neuropsychological assessments at T1, T2, and T3 were part of the standard clinical evaluations for PwTLE undergoing TLR. All data used in this study were obtained from these routine assessments and analyzed retrospectively.

The ethics committee of the University of Münster approved the study protocol (no. 024‐229‐f‐S) and granted a waiver for patient consent in accordance with North Rhine‐Westphalia's health data protection legislation.

### 2.2 Materials

The VLMT,^23^ a German adaptation of the Rey Auditory Verbal Learning Test,^24^ was used in neuropsychological assessments at T1, T2, and T3 to evaluate verbal memory. In German‐speaking regions, the VLMT is regarded as a standard diagnostic tool for assessing verbal memory.^25^, ^26^ In the VLMT, participants are tasked with learning 15 words (the original list) across five oral presentation trials, each followed by immediate recall. After the fifth trial, an interference list of 15 new words is presented, which must be recalled without mentioning the original list. Immediate free recall of the original list follows the interference task and is repeated after a 30‐min delay. Finally, participants must recognize the original 15 words from a selection of 50 words in a forced‐choice (“yes/no”) recognition format. In our study, the 30‐min delayed recall (Trial 7) was used to assess verbal memory function in PwTLE, because it reflects verbal learning and long‐term recall. A deficit in this parameter may arise from impaired learning or reduced recall. Both indicate dysfunction in the language‐dominant temporal lobe.^23^

In total, there are three parallel versions of the VLMT available. Retest reliability analyses based on patient data with an average retest interval of 8–12 months have demonstrated only minimal practice effects for the parameter Trial 7 when administering alternate test versions.^23^

### 2.3 Statistical analyses

Statistical analyses were conducted and graphically presented with IBM SPSS Statistics version 28^27^ and R Studio version 4.4.1.^28^

Raw data from VLMT Trial 7 at T1, T2, and T3 were converted into age‐corrected *z*‐scores using the mean and SD of the test's normative reference groups of healthy controls.^23^ This method was chosen because the VLMT's percentile norms offer limited differentiation in the extreme performance range. Using *z*‐scores therefore provided a more sensitive metric and helped minimize floor effects, which are known to obscure potential postoperative decline in epilepsy surgery.^29^, ^30^ PwTLE with a *z*‐score of <−1 were classified as impaired.^10^ At the group level, to analyze changes in verbal memory function across time points, a repeated measures analysis of variance (RM‐ANOVA) was performed with VLMT Trial 7 as the dependent variable, time point (T1, T2, T3) as the within‐subject factor, and resected hemisphere (language‐dominant vs. language‐nondominant) as the between‐group factor. Post hoc comparisons were conducted to identify significant mean differences between groups and/or time points. At the individual level, frequencies of significant change in verbal memory function across the three time points were calculated for the language‐dominant and language‐nondominant resected groups. Differences in verbal memory function between T1 and T2 and between T2 and T3 were assessed using the *z*‐score change (*z*‐score T2 minus *z*‐score T1; *z*‐score T3 minus *z*‐score T2). A significant decline in verbal memory was defined as a *z*‐score change of <−1, whereas a significant improvement was defined as a *z*‐score change of >1 (which displays a change of 1 SD of the verbal memory test's normative sample) based on previous studies' rationales.^10^ This cutoff was chosen because previous studies in epilepsy surgery cohorts have shown that a *z*‐score change of ±1 approximates the midpoint between more conservative thresholds based on a reliable change index with a 90% confidence interval and most liberal thresholds based on 80% confidence intervals,^31^ thereby striking a balance between sensitivity and specificity in classifying meaningful change. We preferred using *z*‐scores over the reliable change index, as the VLMT's reliable change indices are validated for the adjustment of practice effects up to 12 months only^23^ and thus may be less appropriately applied to the longer time intervals in our study.

To investigate the influence of clinical factors on late change in verbal memory (*z*‐score T3 minus *z*‐score T2) following an initial decline (*z*‐score T2 minus *z*‐score T1 < −1) in the language‐dominant resected group, participants who initially declined were classified into subgroups based on their subsequent memory changes. The following subgroups were established and analyzed: PwTLE who declined again from T2 to T3 (i.e., Double Decliners) compared with those who improved or remained stable between T2 and T3 (i.e., Single Decliners), and PwTLE who showed late improvement after initial decline (i.e., Rebounders) compared with those who either continued to decline or remained unchanged between T2 and T3 (i.e., Non‐Rebounders). To clarify the definition of the subgroups, we would like to state that the Single Decliners partially overlap with the Rebounders, whereas the Double Decliners partially overlap with the Non‐Rebounders. Analyses were conducted in two distinct subgroup contrasts, comparing Double Decliners with Single Decliners and, separately, Rebounders with Non‐Rebounders. Based on our hypotheses, the following six clinical factors were compared between these respective subgroup pairs: (1) change in antiseizure medication drug load from T2 to T3 (calculated using the defined daily doses established by the World Health Organization^32^), (2) seizure outcome (Engel IA vs. Engel worse than IA),^33^ (3) extent of resection (hippocampus resected vs. not resected), (4) histopathological or MRI evidence of MTS (yes vs. no), (5) age at onset, and (6) sex (male vs. female).

## 3 RESULTS

### 3.1 Sample characteristics

The final sample comprised 169 PwTLE. The clinical and demographic data of the sample are presented in Table 1.

**TABLE 1:** Clinical and demographic data.

Variable	Level	Value
Age (T1)	Years	34.92 ± 15.21
Sex	Female	68 (40.24)
Age at onset	Years	17.35 ± 12.03
ASM, defined daily doses^a^		
T1	Quantity	2.40 ± 1.18
T2		2.32 ± 1.22
T3		2.03 ± 1.33
Seizure outcome		
T2	Engel IA	112 (66.27)
T3		93 (55.03)
Prior resection^b^		7 (4.14)
Extent of TLR		
HC resected^c^		125 (73.97)
HC preserved		44 (26.04)
Resection type		
Anterior TLR^d^		101 (59.76)
Lesionectomy		36 (21.30)
Apical TLR^e^		22 (13.02)
Selective amygdalohippocampectomy		10 (5.92)
Etiology		
MTS		84 (49.70)
Tumor		36 (21.30)
MCD		17 (10.10)
Cavernoma		11 (6.51)
Encephalocele		11 (6.51)
Nonlesional		6 (3.55)
Other^f^		4 (2.37)

*Note*: Data are presented as mean ± SD or *n* (%). Engel IA indicates completely seizure‐free.

Abbreviations: ASM, antiseizure medication; HC, hippocampus; MCD, malformations of cortical development; MTS, mesial temporal sclerosis; T1, preoperative; T2, 6 months postoperative; T3, 24 months postoperative; TLR, temporal lobe resection.

^a^ World Health Organization.^32^

^b^ Language‐dominant (*n* = 4); language‐nondominant (*n* = 3); procedure was performed before index TLR.

^c^ Including resection of hippocampal head only.

^d^ Including subtotal temporal lobe resection (*n* = 1).

^e^ Tailored hippocampus‐sparing resection of the apex of the temporal lobe.

^f^ Encephalitis (*n* = 3); lesion not otherwise specified (*n* = 1).

Comparison analyses of missing VLMT data for the initial sample regardless of the inclusion and exclusion criteria revealed that the noncompleters were significantly older (*p* = .01) and had lower preoperative verbal memory performance (*p* = .01), whereas sex (*p* = .84) and resected hemisphere (*p* = .11) did not differ between groups (see Table S1). Comparable results were found for the subsample after applying our exclusion criteria (see Table S2). Missing data for memory testing were most frequent at T3 (*n* = 21). Therefore, we reexamined the reasons for nonattendance or nonparticipation of PwTLE who otherwise met all other inclusion criteria. Twelve of 21 did not return to follow‐up (no response [*n* = 2], refused by patient [*n* = 3], COVID/quarantine [*n* = 3], other medical issues [*n* = 4]), seven of 21 did not undergo memory testing (other medical issues [*n* = 2], organizational issues [*n* = 5]), and two of 21 underwent memory testing at an earlier time point based on epileptological recommendation.

### 3.2 Group‐level and individual analyses

**FIGURE 1:**
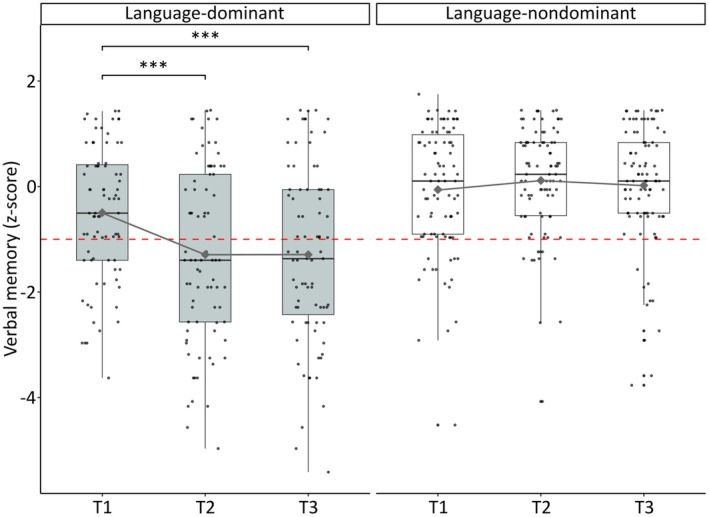
Time course of verbal memory function by resected hemisphere. The dashed red line indicates the cutoff score (*z* < −1) for impaired verbal memory function. Diamonds represent group means at each time point. T1, preoperative; T2, 6 months postoperative; T3, 24 months postoperative. ***Post hoc test, *p* < .001.

Seventy‐nine TLRs were performed in the language‐dominant and 90 in the language‐nondominant hemisphere. At the group level, RM‐ANOVA revealed significant main effects for resected hemisphere with better verbal memory function in the language‐nondominant group compared to the language‐dominant resected group (*F*_1, 167_ = 33.65, *p* < .001, *η* ^2^ = .13) and time point (*F*_2, 321_ = 10.30, *p* < .001, *η* ^2^ = .01). Notably, a significant interaction effect was observed between time point and resected hemisphere (*F*_2, 321_ = 19.49, *p* < .001, *η* ^2^ = .03; see Figure 1). Post hoc tests showed a significant decline in verbal memory in the language‐dominant resected group between T1 and T2 (*p* < .001, *p*_corr_ < .001, *d* = .54) and between T1 and T3 (*p* < .001, *p*_corr_ < .001, *d* = .54). No significant difference was found between T2 and T3 in this group. The language‐nondominant resected group showed no significant changes between time points.

The results of individual changes in verbal memory function for the language‐dominant and language‐nondominant resected group are presented in Table 2. The number of PwTLE classified as “changed” using the reliable change index provided by the VLMT manual^23^ was nearly identical to the number using our approach, which applied 1 SD (based on *z*‐scores) as a criterion for significant change. Only two of 169 PwTLE were classified differently based on the reliable change index.

**TABLE 2:** Significant change in verbal memory function between T1 and T2 and between T2 and T3.

Language‐dominant, *n* = 79					Language‐nondominant, *n* = 90				
		T2–T3					T2–T3		
		↑, *n* = 9	↔, *n* = 54	↓, *n* = 12			↑, *n* = 12	↔, *n* = 61	↓, *n* = 17
T1–T2	↑, *n* = 5	0 [0%]	3 [60%]	2 [40%]	T1–T2	↑, *n* = 13	1 [8%]	8 [62%]	4 [31%]
	↔, *n* = 37	1 [3%]	31 [84%]	5 [14%]		↔, *n* = 67	6 [9%]	48 [72%]	13 [19%]
	↓, *n* = 37	8 [22%]	24 [65%]	5 [14%]		↓, *n* = 10	5 [50%]	5 [50%]	0 [0%]

*Note*: Total values represent the absolute number of cases. Due to the study's focus on the course following initial verbal memory changes, the percentages in square brackets refer to the rows of the table. Values that do not add up to 100 were rounded. Percentages for the entire groups (language‐dominant and language‐nondominant) are provided in Table S3.

Abbreviations: ↑, improvement; ↓, decline; ↔, no change; T1, preoperative; T2, 6 months postoperative; T3, 24 months postoperative.

### 3.3 Postoperative course after initial decline (T1–T2) in language‐dominant resected PwTLE

**FIGURE 2:**
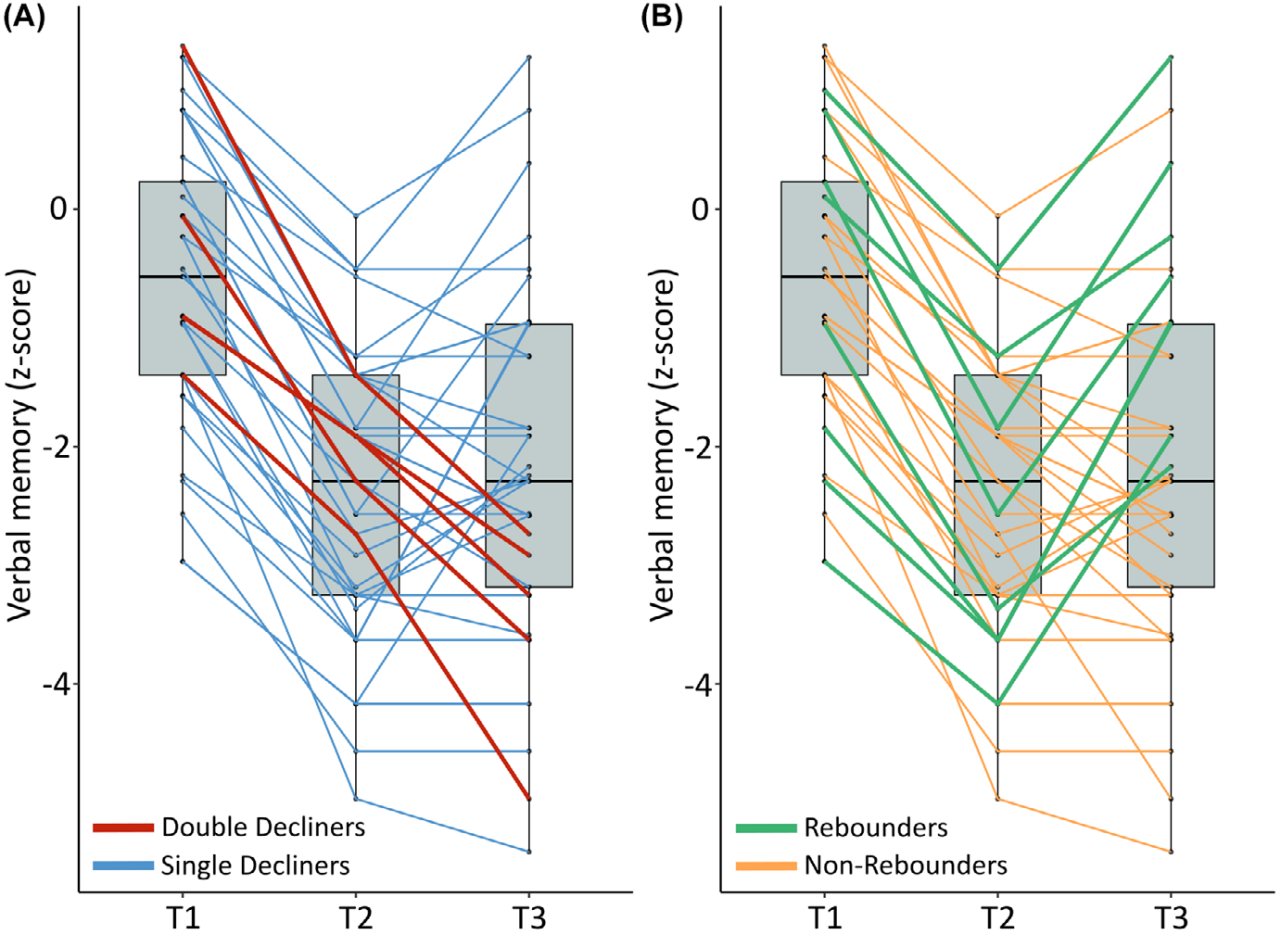
Individual postoperative trajectories following initial verbal memory decline in language‐dominant resected people with temporal lobe epilepsy. (A) Verbal memory trajectories for Double Decliners and Single Decliners. (B) Verbal memory trajectories for Rebounders and Non‐Rebounders. T1, preoperative; T2, 6 months postoperative; T3, 24 months postoperative.

Among the 37 individuals in the language‐dominant resected group who exhibited a significant initial verbal memory decline, five were classified as Double Decliners, whereas 32 were categorized as Single Decliners (see Figure 2A). Additionally, eight individuals were identified as Rebounders, compared with 29 Non‐Rebounders (see Figure 2B).

**FIGURE 3:**
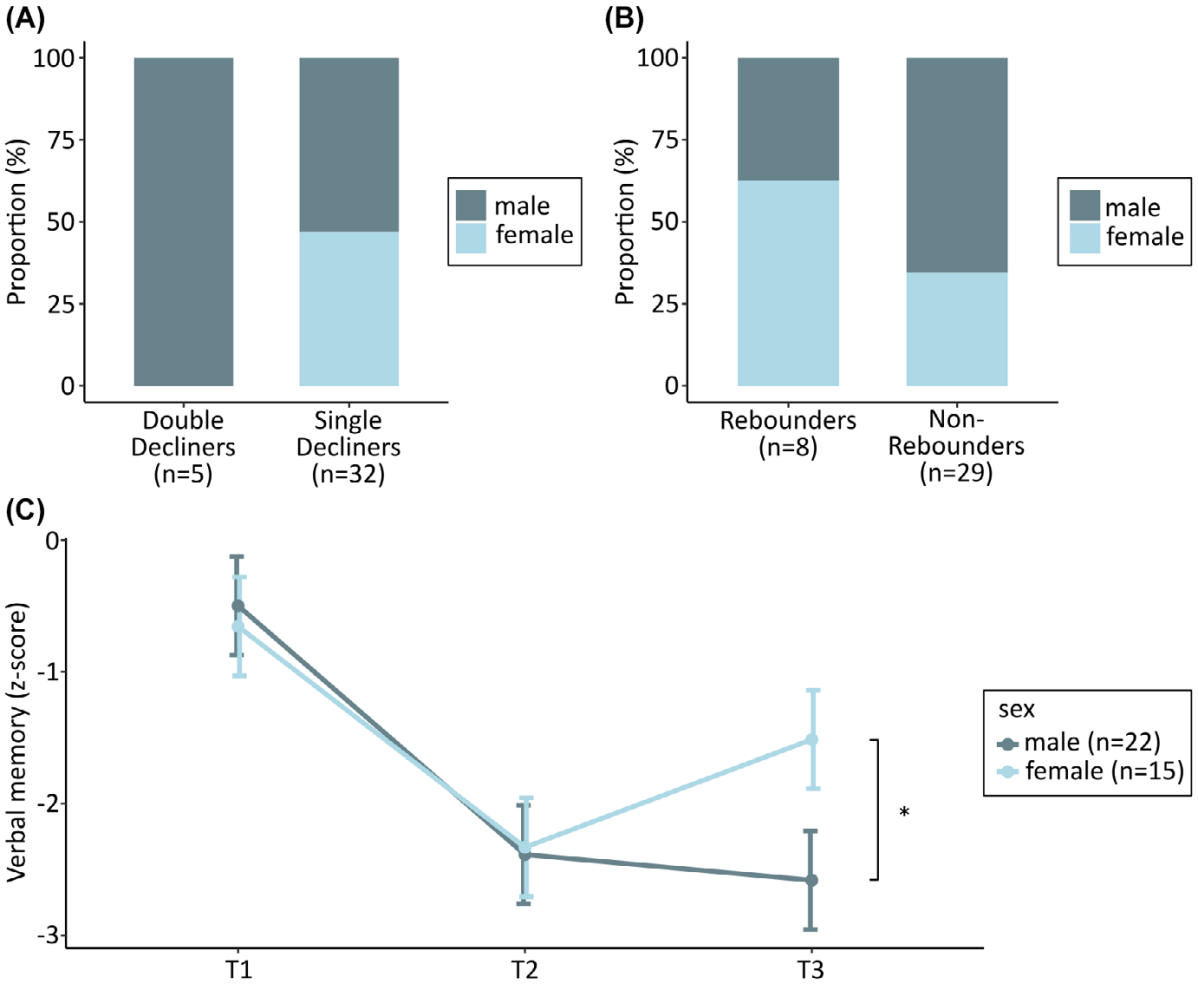
Sex differences in postoperative verbal memory trajectories. (A) Proportion of males and females among Double Decliners (decline from T1 to T2 and from T2 to T3) and Single Decliners (decline from T1 to T2 and no change or improvement from T2 to T3). (B) Proportion of males and females among Rebounders (decline from T1 to T2 and improvement from T2 to T3) and Non‐Rebounders (decline from T1 to T2 and no change or further decline from T2 to T3). (C) Verbal memory trajectories for language‐dominant resected males and females with initial decline between T1 and T2 (*n* = 37); error bars represent standard errors. *Post hoc test, *p* < .05. T1, preoperative; T2, 6 months postoperative; T3, 24 months postoperative.

Preliminary subgroup analyses showed that all Double Decliners were male. By contrast, the Single Decliners exhibited a more balanced sex distribution (male, *n* = 17; female, *n* = 15; see Figure 3A and Table S4). Despite small subgroup sample sizes, descriptive examination indicated that the Rebounders consisted of more females (male, *n* = 3; female, *n* = 5), whereas the Non‐Rebounders included more males than females (male, *n* = 19; female, *n* = 10; see Figure 3B and Table S5). Due to these findings, an additional subsequent RM‐ANOVA was conducted within the language‐dominant resected group for participants who showed a significant initial verbal memory decline between T1 and T2 (*n* = 37). VLMT Trial 7 served as the dependent variable, with time point (T1, T2, T3) as the within‐subject factor and sex (male vs. female) as the between‐group factor, to examine potential sex‐specific postoperative trajectories following the initial verbal memory decline. This revealed a significant main effect of time point (*F*_2, 56_ = 63.06, *p* < .001, *η* ^2^ = .27) but no significant main effect of sex (*F*_1, 35_ = .69, *p* = .41, *η* ^2^ = .02). Notably, there was a significant interaction between time point and sex (*F*_2, 56_ = 7.46, *p* < .001, *η* ^2^ = .04). Post hoc tests showed a significant sex difference at T3 only, with females demonstrating superior verbal memory function compared with males (*p* = .04, *d* = .79; see Figure 3C). No significant differences were found between males and females at T1 or T2. These findings suggest that, following an initial decline, a significant late improvement in memory was observed in the female group (*p* = .01, *p*_corr_ = .04, *d* = .63), whereas memory scores in the male group remained stable at a lower performance level (*p* = .41, *p*_corr_ = 1.00, *d* = .14). Memory decline between T1 and T2 followed a parallel course in both sexes.

Preliminary subgroup analyses of the clinical factors are summarized in Tables S4 and S5 and Figure S2. Double Decliners showed a significantly greater reduction in drug load between T2 and T3 than Single Decliners, whereas Rebounders did not differ from Non‐Rebounders in terms of drug load change. Other variables—including seizure outcome, extent of resection, presence of MTS, and age at onset—did not show any significant differences between the groups (see Tables S4 and S5).

## 4 DISCUSSION

In this study, we investigated the dynamics of verbal memory function in PwTLE following TLR beyond the initial postoperative phase, comparing resections in the language‐dominant versus the language‐nondominant resected group. At the group level, we observed a significant decline in verbal memory after language‐dominant TLR, whereas no significant change was found after language‐nondominant TLR. Specifically, the language‐dominant resected group exhibited a significant initial decline in verbal memory 6 months after surgery, but no further change was detected at the group level 24 months postoperatively. At the individual level, both the language‐dominant and language‐nondominant resected groups showed dynamic patterns, including decline, stability, and even improvement in verbal memory function. We also examined predictors of late verbal memory changes in the language‐dominant resected group following an initial decline. As hypothesized, our analysis revealed sex differences in the trajectory of verbal memory performance^19^, ^20^, ^21^; females demonstrated greater late recovery of verbal memory function than did males after an initial decline. To the best of our knowledge, this is the first study to identify a sex‐specific difference in verbal memory recovery following an initial postoperative decline in the language‐dominant resected group.

### 4.1 Postoperative verbal memory courses at the group level

The postoperative decline in verbal memory function observed in language‐dominant resected PwTLE represents an expected pattern and is consistent with previous studies.^6^, ^11^, ^13^ Although this does not constitute a novel finding, confirming this well‐established association within our cohort was an important initial step to ensure the validity of our subgroup analyses. Nevertheless, our results thus provide further evidence for the critical role of the left temporal lobe in verbal memory function, underscoring the heightened risk of verbal memory decline for PwTLE undergoing language‐dominant TLR.^14^ The specificity of this finding is reinforced by the relative stability of verbal memory function in language‐nondominant resected PwTLE, which aligns with earlier research^13^, ^34^ and suggests a low risk of verbal memory decline following language‐nondominant TLR.^14^

### 4.2 Postoperative verbal memory courses at individual level

Although no late change was observed at the group level, the postoperative time course of verbal memory function proved to be more complex when analyzed at the individual level. This complexity was reflected in the findings that verbal memory performance fluctuated across time points in both the language‐dominant and language‐nondominant resected groups. Several studies have highlighted similar fluctuations, showing significant heterogeneity in verbal memory outcomes within both groups, with changes ranging from deterioration to notable improvement.^10^, ^35^ These findings emphasize that individual changes do not always mirror group‐level trends.^11^ Notably, double improvement was rare and found exclusively in PwTLE and resections in the language‐nondominant hemisphere (1.11% of the language‐nondominant resected group), whereas double declines were limited to the language‐dominant resected group (6.33% of the language‐dominant resected group), illustrating that these contrasting patterns can occur throughout the postoperative course. Our findings thus extend the observations reported by Helmstaedter et al.,^10^ who documented rare cases of double declines and improvements across a mixed language‐dominant and language‐nondominant resected sample (our results: 2.96% double decline, .59% double improvement; Helmstaedter et al.^10^: .62% double decline, 1.24% double improvement) by suggesting a possible association with the resected hemisphere (see Figure S3).

Contrary to the principle often reported in the literature—that individuals with lower baseline cognitive abilities are less likely to experience further decline^36^, ^37^, ^38^—our individual‐level findings show that verbal memory decline can still occur even in PwTLE with low baseline performance (see Figure S3), thereby challenging this assumption. A plausible explanation for this finding may be that we used *z*‐scores based on the VLMT normative sample's mean and SD (see Section 2.3), which allowed us to mitigate test‐inherent floor effects. This methodological choice is relevant, as previous studies showed that floor effects can obscure postoperative memory decline in epilepsy surgery.^29^, ^30^ Furthermore, it could be argued that the use of reliable change indices—commonly employed to determine significant changes (e.g., Baxendale & Thompson^37^)—may have been advantageous over the criterion of 1 SD. To address this idea, we additionally calculated significant changes using the VLMT's 90% reliable change index, which adjusts for practice effects.^23^ Critically, the reliable change index identified nearly identical PwTLE exhibiting significant changes as the results using 1 SD based on *z*‐scores, with only two PwTLE being classified differently.

### 4.3 Sex effects of verbal memory change after initial decline in language‐dominant resected PwTLE

In line with the differing sex ratios observed in our subgroups—namely, higher rates of females among Rebounders and higher rates of males among Double Decliners—we found preliminary evidence for sex‐dependent differences in verbal memory recovery, with late improvement specifically seen in females following an initial decline in language‐dominant resected PwTLE. To our knowledge, this represents the first report of such findings in the existing literature, although the findings should be interpreted with caution given the small subgroup size. These observed differences may reflect sex‐specific processes of neural recovery, adaptation, or the influence of external factors (e.g., social support) that evolve over time. Our results align with prior studies that have demonstrated sex‐related differences, with females showing better verbal memory outcomes following epilepsy surgery.^19^, ^20^ However, it is important to note that previous research usually focused on a single postoperative outcome time point,^19^, ^20^ thereby overlooking the dynamic nature of postoperative verbal memory trajectories. Notably, Bjørnaes et al.^39^ assessed memory functions at three time points and reported that males exhibited an initial decline in verbal long‐term memory after left hemispherical TLR, which then remained stable, whereas females showed neither an early nor late decline. In our study, the sex difference was observed only at 24 months postoperatively, possibly because we focused on PwTLE who had already shown a decline at 6 months, which may potentially have masked earlier sex differences. Notably, considering the total sample of dominant‐resected PwTLE (*n* = 79), we only found a significant main effect of time point (*p* < .001) but no differences between males and females (*p* = .31), and no significant interaction effect (*p* = .31; see Table S6), suggesting a specific sex effect for postoperative trajectories after an initial verbal memory decline only. Overall, our results support the general hypothesis that females tend to be more resistant to the neurocognitive impact of epilepsy surgery (see overview in Lorkiewicz et al.^40^).

As a hypothesis for the observed difference in verbal memory function between males and females at 24 months postoperatively, it may be speculated that at 6 months after surgery, the immediate effects of the procedure (e.g., loss of neural tissue, early stages of neural reorganization, emotional disturbance) are still dominant, although recovery may continue beyond this point.^36^ Differential, sex‐specific trajectories after this initial phase may arise from hormonal differences that influence neuroplasticity,^41^, ^42^, ^43^, ^44^ potentially affecting both the extent and direction of functional recovery. At the cognitive level, sex‐specific late recovery may be influenced by the use of effective memory encoding strategies, such as semantic clustering, which is more commonly employed by females.^36^, ^45^, ^46^ Evidence suggests that even when the degree of hippocampal pathology is comparable across sexes, females often demonstrate superior encoding efficiency.^20^ We speculate that such mechanisms may emerge or become more effective after the initial postoperative phase, offering a potential explanation for the sex differences, which appeared only later in the course. However, this hypothesis requires further confirmation, including a more detailed consideration of PwTLE's memory encoding strategies.

### 4.4 Role of additional clinical factors in verbal memory change after initial decline in language‐dominant resected PwTLE

We found a greater reduction in drug load among Double Decliners than Single Decliners, which was unexpected, because previous studies have reported better cognitive functioning in people with epilepsy who have a lower drug load.^47^ This finding was paralleled by an observed trend toward better seizure outcomes among PwTLE who experienced further late decline (Double Decliners) or no late recovery (Non‐Rebounders) following an initial decline in verbal memory, which is in contrast to previous studies.^30^, ^48^ Other investigations have reported that seizure outcome is not a significant predictor of verbal memory decline after TLR.^6^, ^9^ However, these studies either did not isolate the group with an initial decline or relied on a single postoperative measurement time point. Another potential explanation for the unexpected findings—namely, that a more extensive resection might increase the likelihood of seizure freedom and greater reduction in drug load but also entail an increased risk of verbal memory decline—was not supported by our data (see Table S4). Neither seizure outcome (*p* = .41) nor change in drug load (*p* = .11) showed an association with the extent of resection (hippocampus‐sparing vs. hippocampus‐including). An additional analysis examining the overall extent of resection (anterior TLR vs. more restricted surgical approaches) likewise revealed no significant group differences (anterior TLR: Double Decliners = 60%, Single Decliners = 62.50%, *p* = 1.00; Rebounders = 50.00%, Non‐Rebounders = 65.50%, *p* = .68). We therefore have no robust explanation for our unexpected findings. Still, because of the limited sample sizes of our subgroups (*n* = 5, *n* = 8, as discussed in Limitations), these results should be interpreted with caution.

### 4.5 Limitations

A notable limitation of this study is its retrospective design, which inherently carries the risk of missing data. Although the postoperative follow‐up rate for people with epilepsy at the Bethel Epilepsy Center is high (85.5%),^49^ data for the T3 assessment were missing for 21 PwTLE who otherwise met all inclusion criteria. Although verbal memory decline was not explicitly cited as a reason for nonattendance by any of these cases, one might argue that PwTLE with a pronounced verbal memory decline after TLR may have been less willing or able to participate in the T3 neuropsychological assessment—or may not have attended the follow‐up at all—potentially resulting in underrepresentation of this subgroup. However, a post hoc analysis found no significant difference (*p* = .64) in verbal memory change from T1 to T2 between those who participated in the T3 assessment and those who did not, which does not support a selection bias related to initial verbal memory decline. Exploring the reasons for missing data in a prospective study design could provide valuable insights into differences between participants who remain engaged and those who drop out. Nonetheless, the retrospective design allowed for the inclusion of a relatively large sample, which ultimately enabled the identification and analysis of distinct, clinically relevant subgroups.

Another limitation of this study is the limited generalizability of its findings due to the strict inclusion and exclusion criteria applied (see Figure S1). Although these criteria ensured a highly homogeneous and well‐controlled sample, allowing for the isolation of specific effects of interest, they may also reduce the representativeness of the results for the broader population of PwTLE. Another restriction of generalizability may come from the finding that all VLMT noncompleters (at T1, T2, or T3) were significantly older and had lower preoperative verbal memory performance than completers. This group thus may be underrepresented in our final sample. However, this does not affect the observed sex‐specific verbal memory trajectories, as the groups did not differ significantly with respect to sex distribution. Nevertheless, our carefully selected sample helped minimize variability stemming from potential confounding factors such as intellectual or learning disabilities, language barriers, or atypical language dominance, thereby strengthening the internal validity of our findings. Finally, the small sample sizes of certain subgroups in our analyses of clinical predictors (see Section 3.3; *n* = 5, *n* = 8) call for caution in interpreting these results.

Notwithstanding its limitations, our study design included standardized memory assessments at fixed time points, enabling a systematic analysis of cognitive change after TLR. This is a significant advantage over previous studies that assessed memory function at only a single postoperative time point or at variable intervals, because it allowed us to capture dynamic verbal memory changes occurring beyond the initial postoperative phase. However, the longitudinal scope of our study remains limited, as longer term follow‐ups may reveal additional changes in verbal memory function.^12^ Future research should build on these findings by incorporating extended follow‐up periods to determine whether the observed effects remain stable or evolve over time, thereby providing a more comprehensive understanding of the time course of verbal memory outcomes after TLR. Achieving this will require maintaining long‐term contact with patients, for example, through online follow‐up assessments to monitor cognitive performance over time. These follow‐ups should also include systematic inquiries into the reasons for missed standardized assessments.

## 5 CONCLUSIONS

This study reveals important insights into the postoperative dynamics of verbal memory function following TLR. Our findings provide a detailed characterization of individual verbal memory trajectories after an initial decline. In contrast to group‐level analyses, where late changes are often obscured, examining outcomes at the individual level is essential for capturing the full range of dynamic responses, including both late memory decline and late recovery. These results underscore the importance of serial neuropsychological evaluations that extend beyond the initial postoperative period, as well as the need to consider potential cognitive fluctuations in patient counseling. Notably, sex emerged as a significant factor influencing late postoperative memory outcomes, with women showing greater recovery after an initial decline. These differing cognitive trajectories suggest preliminary evidence for sex‐dependent differences in memory recovery following epilepsy surgery. In sum, our findings support the necessity of personalized counseling in the context of epilepsy surgery, using this knowledge to better inform PwTLE about potential risks and recovery prospects.

## AUTHOR CONTRIBUTIONS

**Pia Langenberg:** Conceptualization; data curation; formal analysis; methodology; visualization; writing—original draft preparation; writing—review & editing. **Lea M. Reisch:** Investigation; data curation; writing—review & editing. **Johanna L. Hopf:** Investigation; data curation. **Florian J. Mücke:** Investigation; data curation; writing—review & editing. **Lea Wemheuer:** Software; visualization; writing—review & editing. **Christian G. Bien:** Conceptualization; supervision; resources; writing—review & editing. **Philip Grewe:** Conceptualization; methodology; supervision; project administration; writing—review & editing.

## CONFLICT OF INTEREST STATEMENT

None of the authors has any conflict of interest to disclose. We confirm that we have read the Journal's position on issues involved in ethical publication and affirm that this report is consistent with those guidelines.

## Supporting information


Figure S1.

Figure S2.

Figure S3.

Table S1.

Table S2

Table S3.

Table S4.

Table S5.

Table S6.

## Data Availability

The data that support the findings of this study are available on request from the corresponding author. The data are not publicly available due to privacy or ethical restrictions.
