# Supplementary material for: Dynamic patterns of verbal memory function after an initial decline following temporal lobe resection against epilepsy: Sex‐specific differences in the postoperative course
Source: Epilepsia. 2026 Feb 14;67(5):2159–70. doi: 10.1002/epi.70144 (PMC13179668; doi:10.1002/epi.70144)
Supplement: Supplementary file 1 — Figure S1. [file EPI-67-2159-s009.docx]

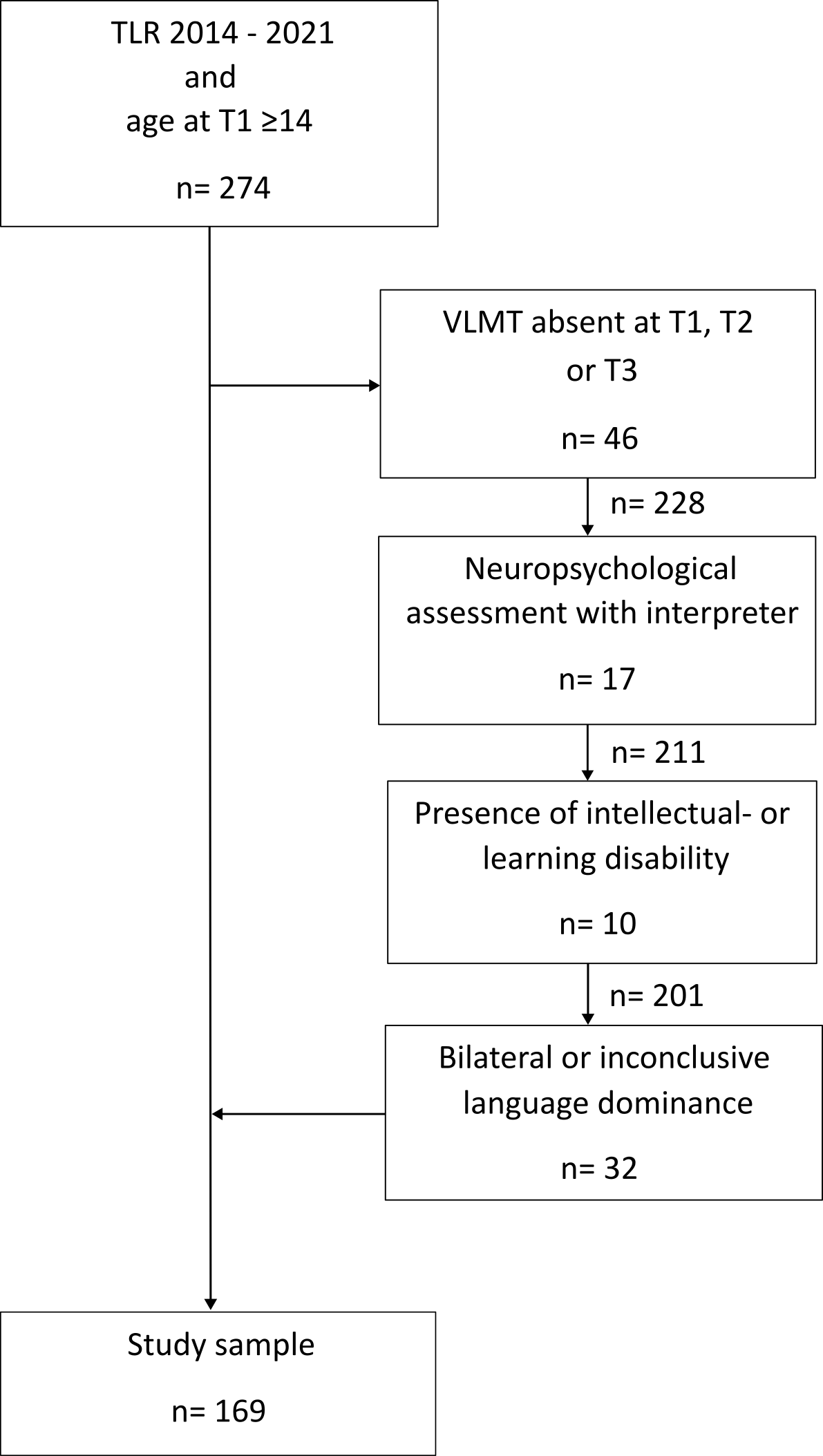


**Figure S1**Inclusion and exclusion criteria of the study sample.
TLR = temporal lobe resection; T1 = preoperative; T2 = six months postoperative; T3 = 24 months postoperative; VLMT = Verbaler Lern- und Merkfähigkeitstest
