# Supplementary material for: Dynamic patterns of verbal memory function after an initial decline following temporal lobe resection against epilepsy: Sex‐specific differences in the postoperative course
Source: Epilepsia. 2026 Feb 14;67(5):2159–70. doi: 10.1002/epi.70144 (PMC13179668; doi:10.1002/epi.70144)
Supplement: Supplementary file 2 — Figure S2. [file EPI-67-2159-s003.docx]

**
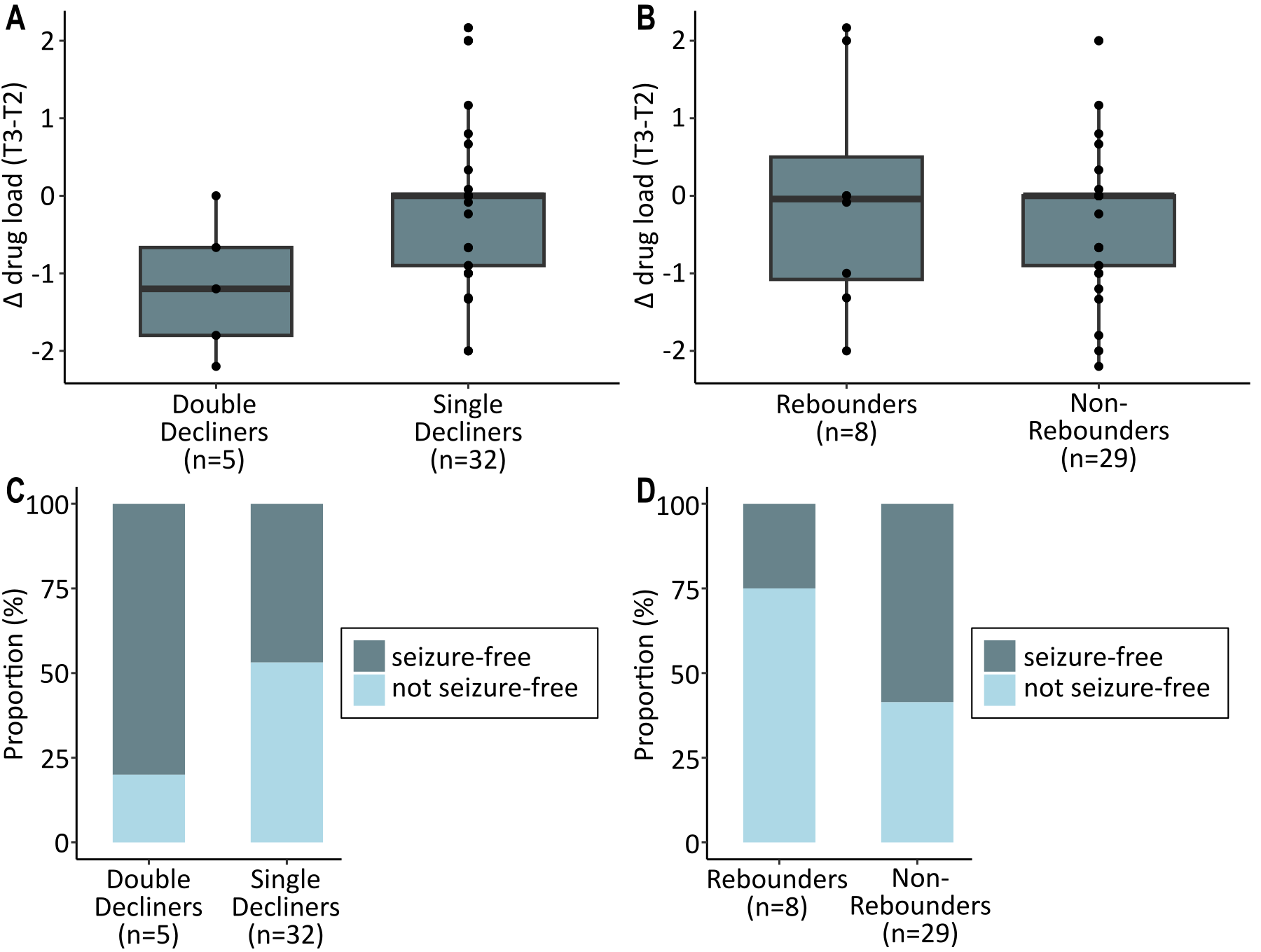
Figure S2**Change in drug load and seizure control at T3 in 38 language-dominant resected PwTLE with an initial verbal memory decline**.**Panel A: Change in anti-seizure medication drug load between T2 and T3 for Double Decliners (decline from T1–T2 and T2–T3) and Single Decliners (decline from T1–T2 and unchanged/improved from T2–T3).
Panel B: Change in anti-seizure medication drug load between T2 and T3 for Rebounders (decline from T1–T2 and improvement from T2–T3) and Non-Rebounders (decline from T1–T2 and unchanged/further decline from T2–T3).
Panel C: Proportion of seizure-free vs. not seizure-free individuals among Double Decliners and Single Decliners at T3.
Panel D: Proportion of seizure-free vs. not seizure-free individuals among Rebounders and Non-Rebounders at T3.
T1 = preoperative; T2 = six months postoperative; T3 = 24 months postoperative
