# Supplementary material for: Dynamic patterns of verbal memory function after an initial decline following temporal lobe resection against epilepsy: Sex‐specific differences in the postoperative course
Source: Epilepsia. 2026 Feb 14;67(5):2159–70. doi: 10.1002/epi.70144 (PMC13179668; doi:10.1002/epi.70144)
Supplement: Supplementary file 3 — Figure S3. [file EPI-67-2159-s008.docx]

**
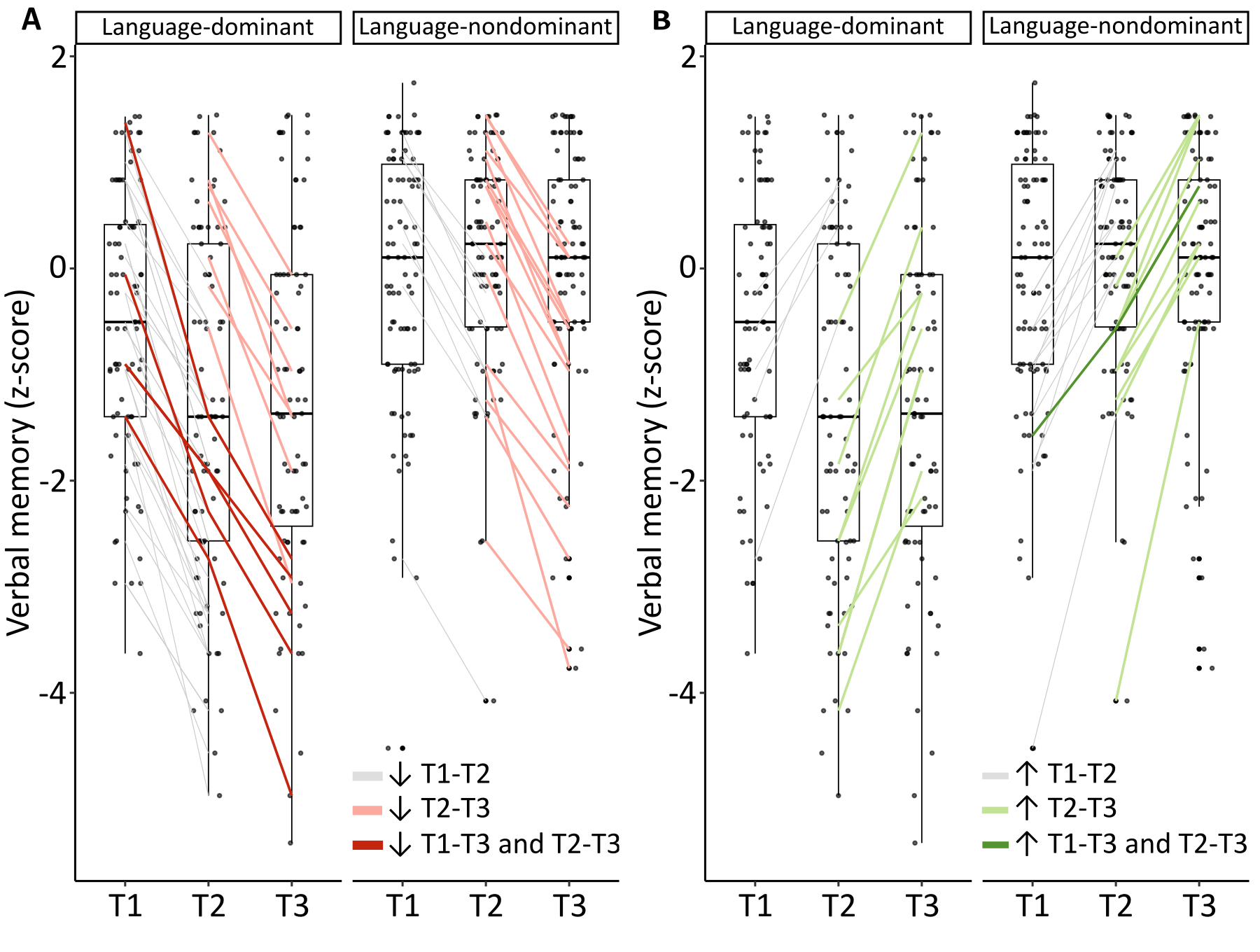
Figure S3**Significant changes in verbal memory function during the postoperative course between language-dominant and language-nondominant resected groups, divided by (A) Decliners and (B) Improvers.
Panel A: Postoperative trajectories of verbal memory decline in language-dominant and language-nondominant resected groups. Gray lines indicate decline between T1–T2, pink lines indicate decline between T2–T3, and red lines indicate decline across both intervals (T1–T2 and T2–T3).
Panel B: Postoperative trajectories of verbal memory improvement in language-dominant and language-nondominant resected groups. Gray lines indicate significant improvement between T1–T2, light green lines indicate improvement between T2–T3, and dark green lines indicate improvement across both intervals (T1–T2 and T2–T3).
T1 = preoperative; T2 = six months postoperative; T3 = 24 months postoperative
