# Supplementary material for: Dynamic patterns of verbal memory function after an initial decline following temporal lobe resection against epilepsy: Sex‐specific differences in the postoperative course
Source: Epilepsia. 2026 Feb 14;67(5):2159–70. doi: 10.1002/epi.70144 (PMC13179668; doi:10.1002/epi.70144)
Supplement: Supplementary file 4 — Table S1. [file EPI-67-2159-s007.docx]

**Table S1.** Comparison analyses of missing VLMT data of the initial sample.

| Variable | Levels | Completers  (*n* = 169) | Non-Completers (*n* = 46) | *p* | ES |
| --- | --- | --- | --- | --- | --- |
| Sex | Female | 68 (40.24) | 23 (50.00) | .24 | 0.08 |
| Age at T1 | Years | 34.92 ± 15.21 | 42.16 ± 15.36^1^ | .01*^2^ | 0.48 |
| Side surgery | Language-dominant | 79 (46.75) | 23 (62.16)^3^ | .11 | 0.12 |
| VLMT T1 | z-score | -0.26 ± 1.25 | -0.89 ± 1.19^4^ | .01*^5^ | 0.51 |

Data are presented as mean ± standard deviation or n (%).
ES = effect size; T1 = preoperative
Fisher’s exact test was used for sex and side of surgery; Cramér’s V was reported for effect size.
A two-sample t-test was conducted for age at T1 and preoperative verbal memory function; Cohen’s d was used for effect size.
**p* ≤ 0.05
^1^ Missing data (*n* = 2)
^2^ Mann–Whitney U test (U = 2716.50, *p* = .01)
^3^ Missing data (*n* = 9)
^4^ Missing data (*n* = 5)
^5^ Mann–Whitney U test (U = 2464.50, *p* = .01)
