# Supplementary material for: Dynamic patterns of verbal memory function after an initial decline following temporal lobe resection against epilepsy: Sex‐specific differences in the postoperative course
Source: Epilepsia. 2026 Feb 14;67(5):2159–70. doi: 10.1002/epi.70144 (PMC13179668; doi:10.1002/epi.70144)
Supplement: Supplementary file 5 — Table S2 [file EPI-67-2159-s005.docx]

**Table S2.** Comparison analyses of missing VLMT data of the subsample after applying our study’s exclusion criteria**.**

| Variable | Levels | Completers (*n* = 169) | Non-Completers (*n* = 30) | *p* | ES |
| --- | --- | --- | --- | --- | --- |
| Sex | Female | 68 (40.24) | 13 (43.33) | .84 | 0.02 |
| Age at T1 | Years | 34.92 ± 15.21 | 42.73 ± 15.81 | .01*^1^ | 0.51 |
| Side surgery | Language-dominant | 79 (46.75) | 19 (63.33) | .12 | 0.12 |
| VLMT T1 | z-score | -0.26 ± 1.25 | -0.95 ± 1.10 | .01*^2^ | 0.56 |

Data are presented as mean ± standard deviation or n (%).
ES = effect size; T1 = preoperative
Fisher’s exact test was used for sex and side of surgery; Cramér’s V was reported for effect size.
A two sample t-test was conducted for age at T1 and preoperative verbal memory function; Cohen’s d was used for effect size.
**p* ≤ 0.05
^1^ Mann–Whitney U test (U = 1790.50, *p* = .01)
^2^ Mann–Whitney U test (U = 1707.50, *p* = .01)
