# Supplementary material for: Dynamic patterns of verbal memory function after an initial decline following temporal lobe resection against epilepsy: Sex‐specific differences in the postoperative course
Source: Epilepsia. 2026 Feb 14;67(5):2159–70. doi: 10.1002/epi.70144 (PMC13179668; doi:10.1002/epi.70144)
Supplement: Supplementary file 6 — Table S3. [file EPI-67-2159-s002.docx]

**Table S3.** Significant change in verbal memory function between T1–T2 and T2–T3.

| Language-dominant (*n* = 79) | | | | |  | Language-nondominant (*n* = 90) | | | | |
| --- | --- | --- | --- | --- | --- | --- | --- | --- | --- | --- |
| T1–T2 | T2–T3 | | | |  | T1–T2 |  | T2–T3 | | |
|  |  | ↑ *n* = 9 | ↔ *n* = 54 | ↓ *n* = 12 |  |  |  | ↑  *n* = 12 | ↔  *n* = 61 | ↓  *n* = 17 |
|  | ↑  *n* = 5 | 0  [0%] | 3  [4%] | 2  [3%] |  |  | ↑  *n* = 13 | 1  [1%] | 8  [9%] | 4  [4%] |
|  | ↔  *n* = 37 | 1  [1%] | 31  [39%] | 5  [6%] |  |  | ↔  *n* = 67 | 6  [7%] | 48  [53%] | 13  [14%] |
|  | ↓  *n* = 37 | 8  [10%] | 24  [30%] | 5  [6%] |  |  | ↓  *n* = 10 | 5  [6%] | 5  [6%] | 0  [0%] |

Total values represent the absolute number of cases. The percentages in square brackets refer to the respective whole group. Values that do not add up to 100 were rounded.

T1 = preoperative; T2 = six months postoperative; T3 = 24 months postoperative; ↑ = improvement; ↔ = no change; ↓ = decline
