# Supplementary material for: Dynamic patterns of verbal memory function after an initial decline following temporal lobe resection against epilepsy: Sex‐specific differences in the postoperative course
Source: Epilepsia. 2026 Feb 14;67(5):2159–70. doi: 10.1002/epi.70144 (PMC13179668; doi:10.1002/epi.70144)
Supplement: Supplementary file 7 — Table S4. [file EPI-67-2159-s004.docx]

**Table S4.** Clinical factors in the language-dominant resected group: comparison between Double Decliners and Single Decliners.

| Variable | Levels | Double Decliners  (*n* = 5) | Single Decliners  (*n* = 32) | *p* | ES |
| --- | --- | --- | --- | --- | --- |
| Sex | Female | 0 (0.00) | 15 (46.88) | .05* | 0.33 |
| Δ drug load | Δ (T3-T2) | −1.17 ± 0.88 | −0.12 ± 1.01 | .04*^, 2^ | -1.05 |
| Seizure outcome | Engel 1A | 4 (80.00) | 15 (46.88) | .17 | 0.23 |
| HC resected¹ | Yes | 3 (60.00) | 27 (84.38) | .23 | 0.20 |
| MTS | Yes | 3 (60.00) | 19 (59.38) | .98 | 0.00 |
| Age at onset | Years | 12.40 ± 10.02 | 15.46 ± 11.01 | .28^3^ | −0.28 |

Data are presented as mean ± standard deviation or n (%).

Engel 1A = completely seizure-free; ES = effect size; HC = hippocampus; MTS = mesial temporal sclerosis; T2 = six months postoperative; T3 = 24 months postoperative.

Fisher’s exact test was used for sex, seizure outcome, HC resected, and MTS; Cramér’s V was reported for effect size.

A two sample t-test was conducted for change in drug load and age at onset; Cohen’s d was used for effect size.

**p* ≤ 0.05
¹ Including resection of hippocampal head only
^2^ Mann–Whitney U test (U = 34.50, *p* = .04)
^3^ Mann–Whitney U test (U = 65.00, *p* = .57)
