# Supplementary material for: Dynamic patterns of verbal memory function after an initial decline following temporal lobe resection against epilepsy: Sex‐specific differences in the postoperative course
Source: Epilepsia. 2026 Feb 14;67(5):2159–70. doi: 10.1002/epi.70144 (PMC13179668; doi:10.1002/epi.70144)
Supplement: Supplementary file 8 — Table S5. [file EPI-67-2159-s006.docx]

**Table S5.** Clinical factors in the language-dominant resected group: comparison between Rebounders and Non-Rebounders.

| Variable | Levels | Rebounders (*n* = 8) | Non-Rebounders (*n* = 29) | *p* | ES |
| --- | --- | --- | --- | --- | --- |
| Sex | Female | 5 (62.50) | 10 (34.48) | .15 | 0.24 |
| Δ drug load | Δ T3-T2 | −0.03 ± 1.49 | −0.33 ± 0.92 | .48^2^ | 0.28 |
| Seizure outcome | Engel 1A | 2 (25.00) | 17 (58.62) | .09 | 0.28 |
| HC resected¹ | Yes | 8 (100.00) | 22 (75.86) | .12 | 0.25 |
| MTS | Yes | 6 (75.00) | 16 (55.17) | .31 | 0.17 |
| Age at onset | Years | 13.25 ± 9.63 | 15.54 ± 10.79 | .60^3^ | −0.21 |

Data are presented as mean ± standard deviation or n (%).
Engel 1A = completely seizure-free; ES = effect size; HC = hippocampus; MTS = mesial temporal sclerosis; T2 = six months postoperative; T3 = 24 months postoperative.
Fisher’s exact test was used for sex, seizure outcome, HC resected, and MTS; Cramér’s V was reported for effect size.
A two sample t-test was conducted for change in drug load and age at onset; Cohen’s d was used for effect size.
¹ Including resection of hippocampal head only
^2^ Mann–Whitney U test (U = 113.50, *p* = .93)
^3^ Mann–Whitney U test (U = 100.50, *p* = .66)
