# Supplementary material for: Dynamic patterns of verbal memory function after an initial decline following temporal lobe resection against epilepsy: Sex‐specific differences in the postoperative course
Source: Epilepsia. 2026 Feb 14;67(5):2159–70. doi: 10.1002/epi.70144 (PMC13179668; doi:10.1002/epi.70144)
Supplement: Supplementary file 9 — Table S6. [file EPI-67-2159-s001.docx]

**Table S6.** Repeated Measures ANOVA of the language dominant resected group (n = 79) with one within-subjects factor (Time: T1, T2, T3) and one between-subjects factor (sex).

|  | Sum of squares | df | Mean square | *F* | *p* |
| --- | --- | --- | --- | --- | --- |
| Time | 29.37 | 1.83 | 16.05 | 19.70 | <.001* |
| Sex | 6.04 | 1 | 6.04 | 1.07 | .31 |
| Time * sex | 1.73 | 1.83 | 0.94 | 1.16 | .31 |
| Residuals time | 114.80 | 140.90 | 0.82 |  |  |
| Residuals sex | 436.50 | 77 | 5.67 |  |  |

T1 = preoperative; T2 = six months postoperative; T3 = 24 months postoperative.
**p* < .05
